# Supplementary material for: The cellular and immunological dynamics of early and transitional human milk
Source: Commun Biol. 2023 May 18;6:539. doi: 10.1038/s42003-023-04910-2 (PMC10195133; doi:10.1038/s42003-023-04910-2)
Supplement: Supplementary file 2 — Supplementary Material [file 42003_2023_4910_MOESM2_ESM.pdf]

SUPPLEMENTARY INFORMATION

The Cellular and Immunological Dynamics of Early and Transitional Human Milk

Cas LeMaster, Stephen H. Pierce, Eric S. Geanes, Santosh Khanal, Staci S. Elliott, Allison B. Scott, Daniel A. Louiselle, Rebecca McLennan, Devika Maulik, Tamorah Lewis, Tomi Pastinen and Todd Bradley

Table of contents

Supplementary Figure 1: Human milk analyte correlations from week 1 and week 2.	Page 2
Supplementary Figure 2: Human milk Ig concentrations differ between samples but not weeks.	Page 3
Supplementary Figure 3. IgG antibodies from human milk and serum differ in Fc-receptor binding characteristics.	Page 4
Supplementary Figure 4: Quality control visualizations.	Page 5
Supplementary Table 1: Summary human milk sample metadata.	Page 6
Supplementary Table 2: Categorizations of soluble analytes.	Page 7
Supplementary Table 3: Sample and cell count frequencies.	Page 8
Supplementary Table 4: Cell type frequency by week.	Page 9
Supplementary Table 5: CD68+ sample cell counts.	Page 10
Supplementary Data 1: Soluble measurements.	XLS file
Supplementary Data 2: Differential gene expression (DGE) across clusters.	XLS file
Supplementary Data 3: CD68+ cells, DGE across timepoints.	XLS file

SUPPLEMENTARY FIGURES

a

Week	Sample ID	1	2	3	4	5	6	7	8	9	10	11	12	13	14	15	16	17	18
Week 1	Soluble	Yes	Yes	Yes	Yes	Yes	Yes	Yes	Yes	Yes	Yes	Yes	Yes	Yes	Yes	Yes	Yes	Yes	Yes
	Cellular	Yes	No	No	Yes	No	Yes	Yes	No	Yes	Yes	Yes	No	No	No	No	No	Yes	Yes
Week 2	Soluble	Yes	No	No	No	Yes	Yes	No	Yes	Yes	Yes	Yes	Yes	Yes	No	Yes	Yes	Yes	Yes
	Cellular	Yes	No	No	No	No	Yes	No	No	Yes	No	No	No	No	No	No	Yes	No	Yes

Week	Sample ID	19	20	21	22	23	24	25	26	27	28	29	30	31	32	33	34	35	36
Week 1	Soluble	Yes	Yes	Yes	Yes	Yes	Yes	Yes	Yes	Yes	Yes	No	Yes	Yes	Yes	No	Yes	Yes	Yes
	Cellular	Yes	No	No	No	No	No	No	No	No	No	No	No	No	No	No	No	No	No
Week 2	Soluble	Yes	No	Yes	Yes	Yes	Yes	Yes	Yes	Yes	Yes	Yes	Yes	Yes	Yes	No	No	No	No
	Cellular	Yes	No	No	No	No	No	No	No	No	No	No	No	No	No	No	No	No	No

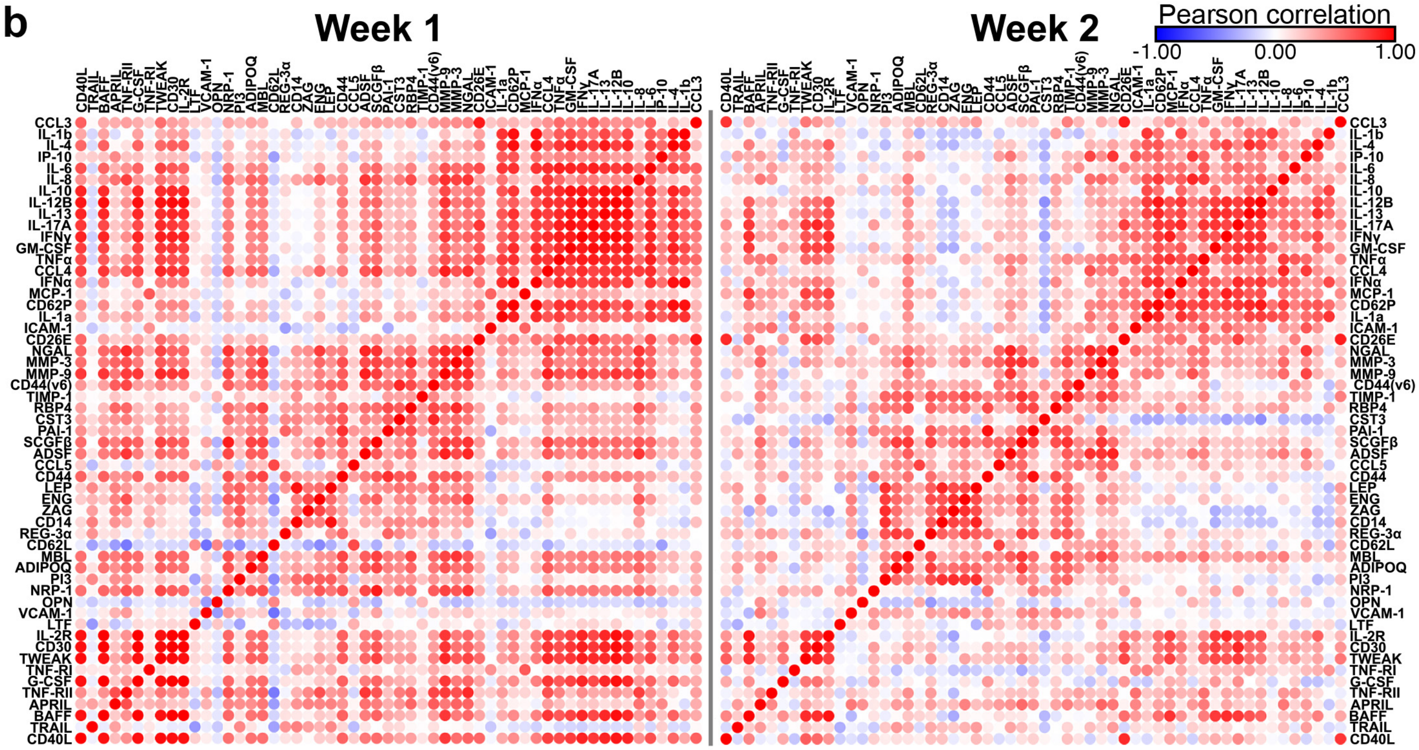

Supplementary Figure 1: Breast milk samples. (**a**) Breast milk samples marked as yes or no if analyzed for soluble and cellular components of the study (**b**) Breast milk analyte correlations. Side-by-side Pearson correlations of 55 measured cytokines, chemokines, growth factors, enzymes, adhesion molecules, and lipocalins in week 1 (n=34) and week 2 (n=26) samples.

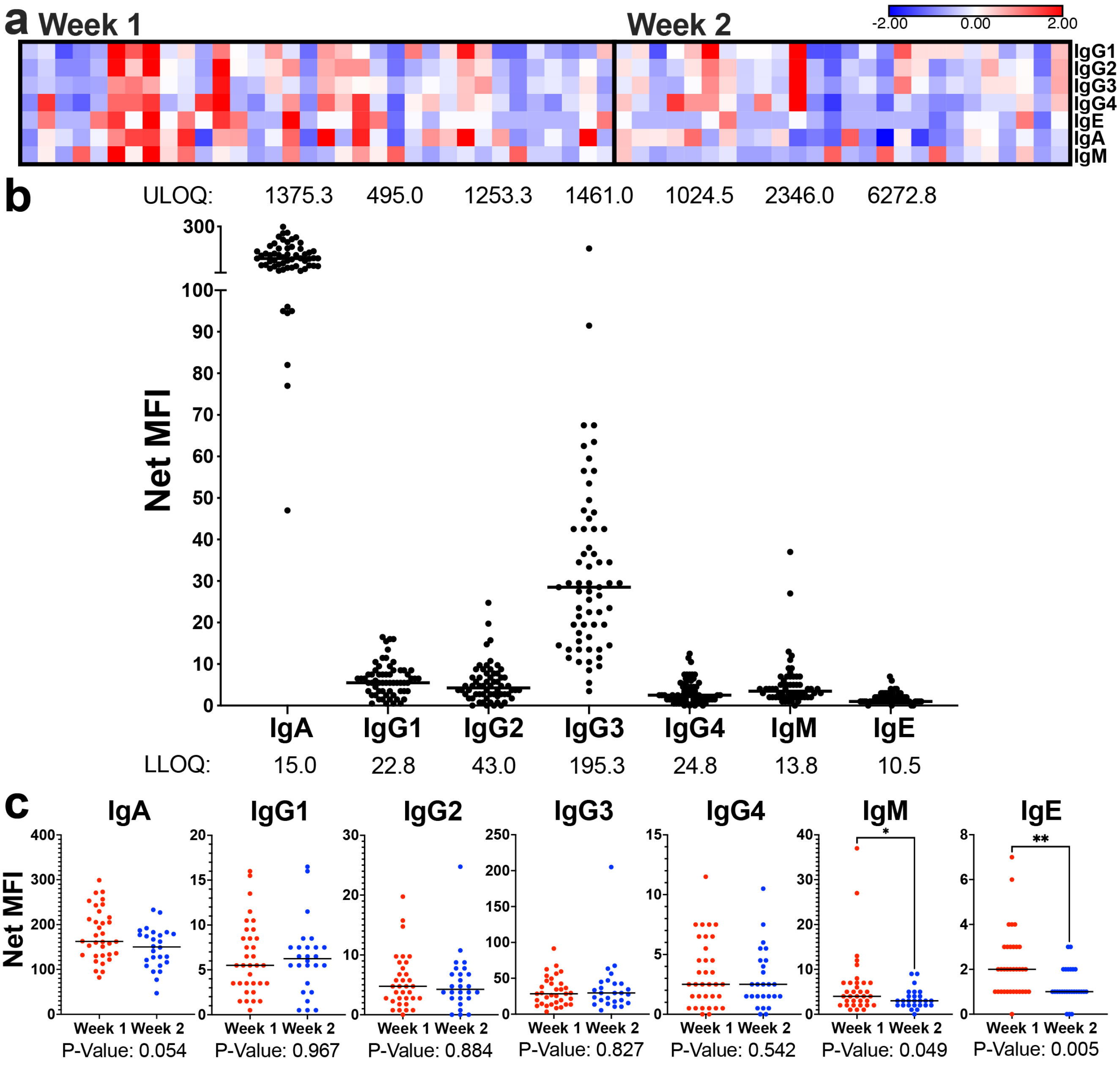

Supplementary Figure 2: Human milk Ig concentrations differ between samples but not weeks. (**a**) Heatmap of z-scores for Ig concentrations from week 1 and week 2 samples. (**b**) Dot plot of immunoglobulin isotypes measured across all samples net MFI values (n=60 samples). Black lines are representative of the median of all samples. Dots are representative of individual samples. Upper limit of quantification (ULOQ) for each Ig above dot plot, lower limit of quantification (LLOQ) for each Ig below dot plot. (**c**) Dot plots of net MFI for immunoglobulin isotypes, separated by week 1 (red) and week 2 (blue) (n=34 samples and 26 samples respectively). Net MFIs are calculated by subtracting MFI of negative control well from the sample well. Black lines are representative of the median of all samples. Dots are representative of individual samples. Significance was determined with Mann-Whitney tests. *, P<0.05, **, P<0.005.

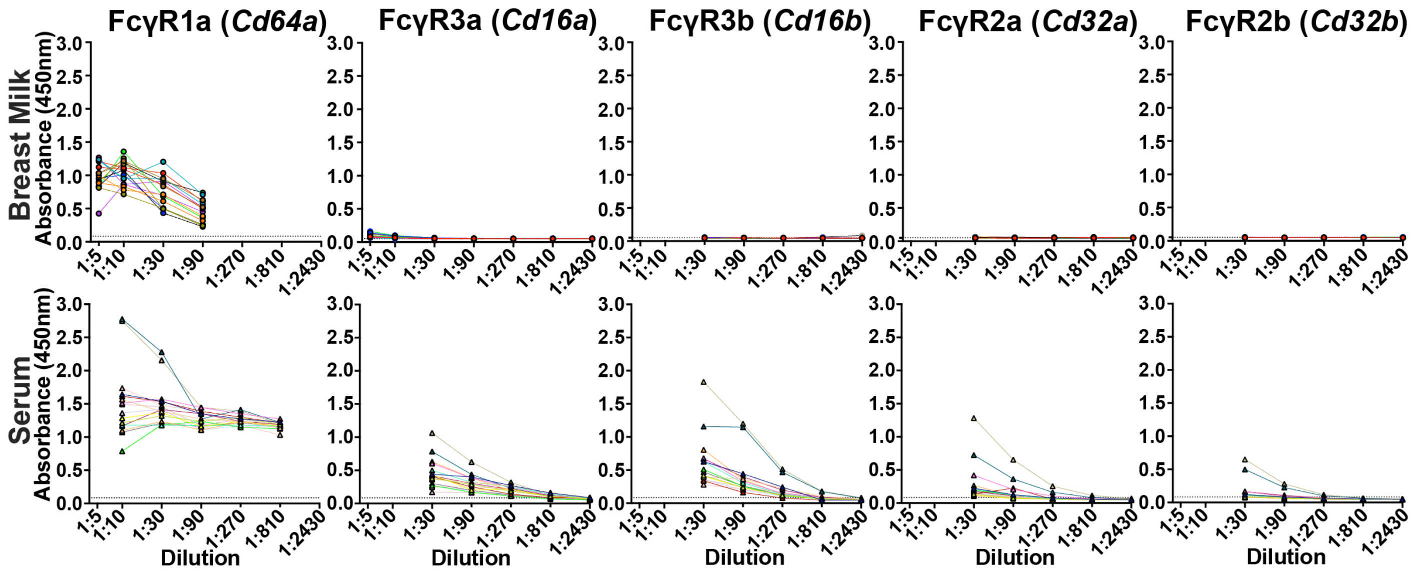

Supplementary Figure 3. IgG antibodies from human milk and serum differ in Fc-receptor binding characteristics. Line graphs of Fc-receptor (FcγR1a, FcγR3a, FcγR3b, FcγR2a, and FcγR2b) responses at different dilutions of human milk and serum measured by ELISA. Serum, n = 16; human milk, n = 16.

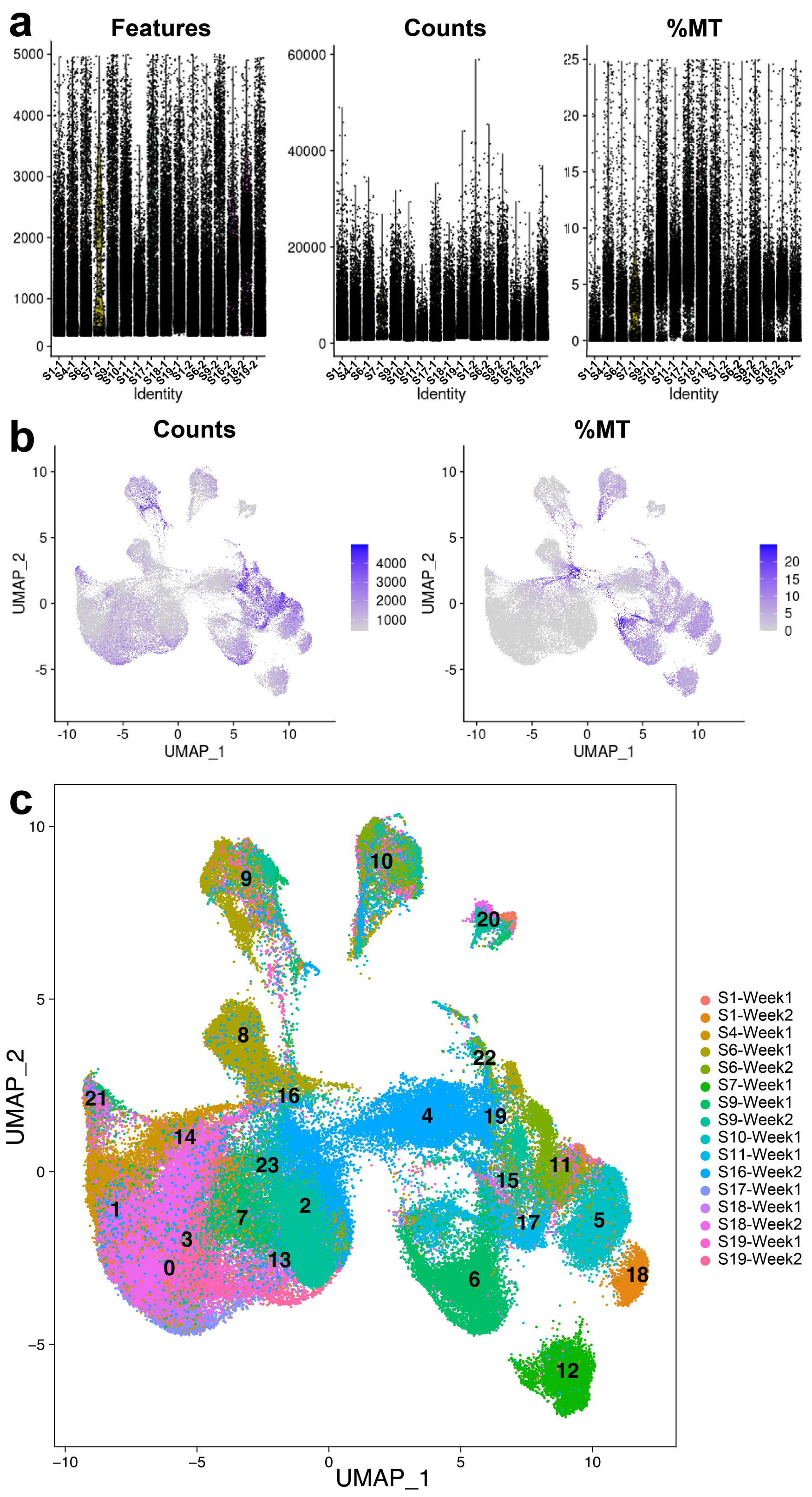

Supplementary Figure 4: Quality control visualizations. (**a**) Violin plots of the number of features (genes) with at least one read, counts (number of reads or UMI), and the percentage of mitochondrial content across all cells. (**b**) UMAPs for the number of features (genes) and the mitochondrial content across clusters. (**c**) UMAP visualizing the distribution of sample-specific cells across clusters.

SUPPLEMENTARY TABLES

Supplementary Table 1: Summary human milk sample metadata.

	**Week 1 (2-7 days, N=36)**	**Week 2 (8-16 days, N=28)**
**Maternal age**	Range 20-42 years old<br>Median 30 years old	Range 20-42 years old<br>Median 28 years old
**Infant age**	Range 2-7 days old<br>Median 4 days old	Range 8-16 days old<br>Median 11 days old
**Gestation**	Range 22-40.2 weeks<br>Median 37 weeks	Range 23.5-40.2 weeks<br>Median 37.1 weeks
**Pregnancy**	Term: 25<br>Preterm: 11	Term: 18<br>Preterm: 10
**Maternal BMI**	Range 17.4-43.9<br>Median 25	
**Race**	White: 29<br>Black or African American: 3<br>American Indian or Alaska Native: 0<br>Asian: 2<br>Native Hawaiian or Other Pacific Islander: 0<br>Multiracial: 1<br>Unknown: 1	White: 22<br>Black or African American: 3<br>American Indian or Alaska Native: 0<br>Asian: 2<br>Native Hawaiian or Other Pacific Islander: 0<br>Multiracial: 1<br>Unknown: 0
**Ethnicity**	Hispanic or Latino: 2<br>Not Hispanic or Latino: 34	Hispanic or Latino: 1<br>Not Hispanic or Latino: 27

Supplementary Table 2: Categorizations of soluble analytes.

Cytokines	Adhesion	Chemokine
IL-1B	CD62P	CCL3
IL-4	ICAM-1	CCL4
IL-6	CD26E	MCP-1
IL-10	CD44 (v6)	CCL5
IL-12B	CD44	IP-10
IL-13	CD14	IL-8
IL-17A	CD62L	**Adipokine**
IFNγ	MBL	RBP4
GM-CSF	NRP-1	ADSF
TNFα	OPN	LEP
IFNα	VCAM-1	ZAG
IL-1a	LTF	ADIPOQ
IL-2R	CD40L	**Enzyme**
CD30	**Immunoglobulin**	MMP-3
TWEAK	IgG1	MMP-9
TNF-RI	IgG2	TIMP-1
G-CSF	IgG3	PI3
TNF-RII	IgG4	**Growth Factor**
APRIL	IgE	SCGFβ
BAFF	IgA	ENG
TRAIL	IgM	REG-3α
**Protease Inhibitor**	**Lipocalin**	**Total protein density**
CST3	NGAL	Protein
PAI-1		

Supplementary Table 3: Sample and cell count frequencies.

Week 1

Type/Donor	S1	S4	S6	S7	S9	S10	S11	S17	S18	S19	Total	Frequency
LC1/LC2	0.0767	0.8418	0.8904	0.1311	0.5005	0.0322	0.0594	0.9215	0.6641	0.6362	0.4754	0.56664938
Macrophage/Monocyte	0.5673	0.0966	0.0644	0.8587	0.4445	0.8986	0.8855	0.0252	0.2115	0.138	0.419	0.35935191
T/NK	0.0616	0.0267	0.0374	0.0084	0.0153	0.0495	0.0364	0.0228	0.0604	0.1904	0.0509	0.04299417
Cell-cycling	0.2881	0.004	0.0002	0.0004	0.0121	0.001	0.0049	0.004	0.0482	0.0052	0.0368	0.01486714
Neutrophils	0.0013	0.0289	0.0022	0.0002	0.0235	0.0006	0.0035	0.0256	0.0116	0.0201	0.0117	0.01025275
B cells	0.005	0.002	0.0054	0.0013	0.0041	0.0182	0.0103	0.0009	0.0042	0.0101	0.0061	0.00588464

Week 2

Type/Donor	S1	S6	S9	S16	S18	S19	Total	Frequency
LC1/LC2	0.20884521	0.04304828	0.76327078	0.39226268	0.8750937	0.9278607	0.54537204	0.40034997
Macrophage/Monocyte	0.76307476	0.69953461	0.13954424	0.54990627	0.04610195	0.02137042	0.36195867	0.20714193
T/NK	0.02316602	0.20826062	0.03297587	0.04056127	0.01264993	0.03324288	0.05621962	0.02813999
Cell-cycling	0	0.0017452	0.05898123	0.00295404	0.04300975	0.00214835	0.01828946	0.00453662
Neutrophils	0.000351	0.00029087	0.00120643	0.00062489	0.02023988	0.01266395	0.00603684	0.01265068
B cells	0.00456301	0.04712042	0.00402145	0.01369085	0.0029048	0.0027137	0.01212338	0.00649384

Week 1

Cluster\Sample	S1	S4	S6	S7	S9	S10	S11	S17	S18	S19	Total	Frequency	Cell type
0	0	332	6	10	118	23	5	4891	495	145	6025	0.0781	LC2
1	25	5272	9	2	160	20	20	1152	742	541	7943	0.103	LC2
2	0	49	78	54	355	15	13	84	92	35	775	0.01	LC2
3	1	45	3	8	112	39	1	721	92	2666	3688	0.0478	LC2
4	1	13	42	15	26	54	43	4	19	38	255	0.0033	Macrophage/Monocyte
5	11	58	4	30	13	7594	32	12	7	14	7775	0.1008	Macrophage/Monocyte
6	16	14	6	24	7273	66	214	6	82	16	7717	0.1	Macrophage/Monocyte
7	3	124	22	35	6130	31	38	79	204	57	6723	0.0871	LC2
8	0	24	5827	1	10	10	1	2	1	2	5878	0.0762	LC2
9	1	486	2977	38	817	52	48	216	197	72	4904	0.0636	LC1
10	49	284	421	46	255	446	134	200	276	1206	3317	0.043	T/NK Cells
11	91	860	13	9	13	68	127	157	349	738	2425	0.0314	Macrophage/Monocyte
12	1	5	0	4615	3	16	3	1	9	7	4660	0.0604	Macrophage/Monocyte
13	24	194	11	17	115	42	26	765	876	341	2411	0.0313	LC2
14	1	2231	8	10	117	19	11	137	247	90	2871	0.0372	LC2
15	309	61	48	12	49	267	732	33	483	48	2042	0.0265	Macrophage/Monocyte
16	6	184	1084	21	376	35	54	37	86	78	1961	0.0254	LC2
17	4	10	1	2	7	15	2105	3	9	9	2165	0.0281	Macrophage/Monocyte
18	15	2	0	1	0	14	1	0	0	0	33	0.0004	Macrophage/Monocyte
19	3	3	611	3	1	8	6	5	8	4	652	0.0085	Macrophage/Monocyte
20	229	42	2	2	201	9	18	35	220	33	791	0.0103	Neutrophils
21	1	307	25	1	390	5	13	225	53	127	1147	0.0149	Cell-cycling
22	4	21	61	7	68	164	38	8	19	64	454	0.0059	B Cells
23	0	0	0	523	5	4	2	0	1	3	538	0.007	LC2
Total	795	10621	11259	5486	16614	9016	3685	8773	4567	6334	77150		
Frequency	0.0103	0.1377	0.1459	0.0711	0.2153	0.1169	0.0478	0.1137	0.0592	0.0821			

Week 2

Cluster\Sample	S1	S6	S9	S16	S18	S19	Total	Frequency	Cell type
0	25	2	12	39	4884	373	5335	0.1049	LC2
1	42	3	25	32	2218	928	3248	0.0639	LC2
2	32	26	4782	5235	98	45	10218	0.2009	LC2
3	35	0	19	39	851	5477	6421	0.1262	LC2
4	1	32	131	8179	70	18	8431	0.1657	Macrophage/Monocyte
5	77	51	8	15	4	5	160	0.0031	Macrophage/Monocyte
6	6	16	34	2	16	1	75	0.0015	Macrophage/Monocyte
7	277	3	246	174	47	20	767	0.0151	LC2
8	10	0	42	124	8	2	186	0.0037	LC2
9	99	14	343	172	173	110	911	0.0179	LC1
10	66	716	246	714	135	294	2171	0.0427	T/NK Cells
11	9	2011	25	55	291	142	2533	0.0498	Macrophage/Monocyte
12	1	10	0	0	1	0	12	0.0002	Macrophage/Monocyte
13	43	90	65	551	468	691	1908	0.0375	LC2
14	20	0	6	32	488	354	900	0.0177	LC2
15	24	207	831	242	83	9	1396	0.0274	Macrophage/Monocyte
16	9	10	153	499	97	206	974	0.0191	LC2
17	0	37	4	14	6	13	74	0.0015	Macrophage/Monocyte
18	2056	1	0	0	0	0	2057	0.0404	Macrophage/Monocyte
19	0	40	8	1173	21	1	1243	0.0244	Macrophage/Monocyte
20	0	6	440	52	459	19	976	0.0192	Neutrophils
21	1	1	9	11	216	112	350	0.0069	Cell-cycling
22	13	162	30	241	31	24	501	0.0098	B Cells
23	3	0	1	8	7	0	19	0.0004	LC2
Total	2849	3438	7460	17603	10672	8844	50866		
Frequency	0.05600991	0.06758935	0.14665985	0.34606614	0.20980616	0.1738686			

Supplementary Table 4: Cell type frequency by week.

Cell Type	Week 1	Week 2
Epithelial cells	47.50%	50.01%
Macrophages	40.43%	34.93%
Monocytes	3.59%	4.56%
T cells	3.47%	3.60%
Dendritic cells	1.31%	2.25%
Neutrophils	0.77%	1.63%
Stem cells	0.65%	0.58%
NK cells	0.48%	0.49%
B cells	0.42%	0.42%
Other	1.37%	1.53%

Supplementary Table 5: CD68+ sample cell counts.

Week 1	Donor	S1	S4	S6	S7	S9	S10	S11	S17	S18	S19	Total
CD68+	Count	434	1011	632	4603	6515	7699	2825	213	920	838	25690
Total Cells	Count	795	10621	11259	5486	16614	9016	3685	8773	4567	6334	77150

Week 2	Donor	S1	S6	S9	S16	S18	S19	Total
CD68+	Count	2110	2333	945	5375	433	170	11366
Total Cells	Count	2849	3438	7460	17603	10672	8844	50866
