# Supplementary material for: The cellular and immunological dynamics of early and transitional human milk
Source: Commun Biol. 2023 May 18;6:539. doi: 10.1038/s42003-023-04910-2 (PMC10195133; doi:10.1038/s42003-023-04910-2)
Supplement: Supplementary file 3 — Description of Additional Supplementary Files [file 42003_2023_4910_MOESM3_ESM.pdf]

Description of Additional Supplementary Files

File name: Supplementary Data 1

File Description: Soluble measurements.

File name: Supplementary Data 2

File Description: Differential gene expression (DGE) across clusters.

File name: Supplementary Data 3

File Description: CD68+ cells, DGE across timepoints.
