# Supplementary material for: The cellular and immunological dynamics of early and transitional human milk
Source: Commun Biol. 2023 May 18;6:539. doi: 10.1038/s42003-023-04910-2 (PMC10195133; doi:10.1038/s42003-023-04910-2)
Supplement: Supplementary file 7 — Reporting Summary [file 42003_2023_4910_MOESM7_ESM.pdf]

nature portfolio

Corresponding author(s): Todd Bradley

Last updated by author(s): Apr 26, 2023

Reporting Summary

Nature Portfolio wishes to improve the reproducibility of the work that we publish. This form provides structure for consistency and transparency in reporting. For further information on Nature Portfolio policies, see our Editorial Policies and the Editorial Policy Checklist.

Statistics

For all statistical analyses, confirm that the following items are present in the figure legend, table legend, main text, or Methods section.

n/a	Confirmed	
☐	☒	The exact sample size (*n*) for each experimental group/condition, given as a discrete number and unit of measurement
☐	☒	A statement on whether measurements were taken from distinct samples or whether the same sample was measured repeatedly
☐	☒	The statistical test(s) used AND whether they are one- or two-sided<br>*Only common tests should be described solely by name; describe more complex techniques in the Methods section.*
☐	☒	A description of all covariates tested
☐	☒	A description of any assumptions or corrections, such as tests of normality and adjustment for multiple comparisons
☐	☒	A full description of the statistical parameters including central tendency (e.g. means) or other basic estimates (e.g. regression coefficient) AND variation (e.g. standard deviation) or associated estimates of uncertainty (e.g. confidence intervals)
☐	☒	For null hypothesis testing, the test statistic (e.g. *F*, *t*, *r*) with confidence intervals, effect sizes, degrees of freedom and *P* value noted<br>*Give P values as exact values whenever suitable.*
☒	☐	For Bayesian analysis, information on the choice of priors and Markov chain Monte Carlo settings
☒	☐	For hierarchical and complex designs, identification of the appropriate level for tests and full reporting of outcomes
☒	☐	Estimates of effect sizes (e.g. Cohen's *d*, Pearson's *r*), indicating how they were calculated

Our web collection on statistics for biologists contains articles on many of the points above.

Software and code

Policy information about availability of computer code

Data collection	N/A
Data analysis	N/A

For manuscripts utilizing custom algorithms or software that are central to the research but not yet described in published literature, software must be made available to editors and reviewers. We strongly encourage code deposition in a community repository (e.g. GitHub). See the Nature Portfolio guidelines for submitting code & software for further information.

Data

Policy information about availability of data

All manuscripts must include a data availability statement. This statement should provide the following information, where applicable:

- Accession codes, unique identifiers, or web links for publicly available datasets
- A description of any restrictions on data availability
- For clinical datasets or third party data, please ensure that the statement adheres to our policy

The accession number for the RNA sequencing data reported in this paper is PRJNA835152

Human research participants

Policy information about studies involving human research participants and Sex and Gender in Research.

Reporting on sex and gender

A total of 37 different females were enrolled in this study for HM. A total of 16 different females, not involved in milk collection, were enrolled for serum immunoglobulin comparisons.

Population characteristics

Population characteristics are provided for the following:
Week 1 (2-7 days, N=36)
Maternal age -
Range 20-42 years old
Median 30 years old
Infant age -
Range 2-7 days old
Median 4 days old
Gestation -
Range 22-40.2 weeks
Median 37 weeks
Pregnancy -
Term: 25
Preterm: 11
Maternal BMI -
Range 17.4-43.9
Median 25
Race -
White: 29
Black or African American: 3
American Indian or Alaska Native: 0
Asian: 2
Native Hawaiian or Other Pacific Islander: 0
Multiracial: 1
Unknown: 1
Ethnicity -
Hispanic or Latino: 2
Not Hispanic or Latino: 34

Week 2 (8-16 days, N=28)
Maternal age -
Range 20-42 years old
Median 28 years old
Infant age -
Range 8-16 days old
Median 11 days old
Gestation -
Range 23.5-40.2 weeks
Median 37.1 weeks
Pregnancy -
Term: 18
Preterm: 10
Maternal BMI -
Range 17.4-43.9
Median 25
Race -
White: 22
Black or African American: 3
American Indian or Alaska Native: 0
Asian: 2
Native Hawaiian or Other Pacific Islander: 0
Multiracial: 1
Unknown: 0
Ethnicity -
Hispanic or Latino: 1
Not Hispanic or Latino: 27

Recruitment

Mothers were enrolled internally through research coordination at Children's Mercy Kansas City as part of an ongoing study reviewed by Children's Mercy's Institutional Review Board (IRB) in accordance with requirements of local governing regulatory agencies including the Department of Health and Human Services (DHHS) and Food and Drug Administration (FDA)

	Codes of Federal Regulations, on the Protection of Human Subjects (45 CFR Part 46 and 2l CFR Part 56, respectively).<br><br>With consideration of neonatal nutrition requirements, low volumes of HM were collected at two different timepoints through electronic pumping, week 1 (2-7 days postnatal) and again at week 2 (8-16 days postnatal).
Ethics oversight	Children's Mercy's Institutional Review Board (IRB)

Note that full information on the approval of the study protocol must also be provided in the manuscript.

Field-specific reporting

Please select the one below that is the best fit for your research. If you are not sure, read the appropriate sections before making your selection.

[x] Life sciences [ ] Behavioural & social sciences [ ] Ecological, evolutionary & environmental sciences

For a reference copy of the document with all sections, see nature.com/documents/nr-reporting-summary-flat.pdf

Life sciences study design

All studies must disclose on these points even when the disclosure is negative.

Sample size	Human milk: 37, Human serum: 16
Data exclusions	Data was only excluded during volcano plot analysis of analytes involved in k means clustering of samples. Here one sample, defining cluster 4, was determined an outlier.
Replication	Platform metrics were validated in comparison to blood serum prior to study assays. Commercial kits and serum or PBMC considerations were applied to human milk in order to maintain consistency and replication in future studies. Biological replicates were processed in singleton for all sequencing and assays.
Randomization	Grouping based on post-natal week of lactation.
Blinding	No blinding was involved in this study.

Reporting for specific materials, systems and methods

We require information from authors about some types of materials, experimental systems and methods used in many studies. Here, indicate whether each material, system or method listed is relevant to your study. If you are not sure if a list item applies to your research, read the appropriate section before selecting a response.

Materials & experimental systems

n/a	Involved in the study	
[x]	[ ]	Antibodies
[x]	[ ]	Eukaryotic cell lines
[x]	[ ]	Palaeontology and archaeology
[x]	[ ]	Animals and other organisms
[x]	[ ]	Clinical data
[x]	[ ]	Dual use research of concern

Methods

n/a	Involved in the study	
[x]	[ ]	ChIP-seq
[x]	[ ]	Flow cytometry
[x]	[ ]	MRI-based neuroimaging
